# Correction to “Typhoid Fever in India: A Growing Concern Requiring Immediate Preventive Efforts”

**DOI:** 10.1002/hsr2.70932

**Published:** 2025-06-20

K. Mehta, M. Joshi, and M. A. Omar, “Typhoid Fever in India: A Growing Concern Requiring Immediate Preventive Efforts,” *Health Science Reports* 7, no. 2 (2024): e1899, https://doi.org/10.1002/hsr2.1899.

In the paragraph 2, “India recorded approximately 10 million cases of typhoid fever in 2021, making it the country with the highest burden of typhoid worldwide” is incorrect and should be, “In 2021, India contributed the highest number of incident cases, totaling 5.4 million (95% UI: 4.1–6.9 million), which represented approximately 58% of global cases. Making it the country with the highest burden”.

Reference: D. Piovani, G. Figlioli, G. K. Nikolopoulos, and S. Bonovas, “The Global Burden of Enteric Fever, 2017‐2021: A Systematic Analysis From the Global Burden of Disease Study 2021,” *EClinicalMedicine* 77 (2024): 102883. https://doi.org/10.1016/j.eclinm.2024.102883.

We apologize for these errors.

